# Mental health analysis of international students using machine learning techniques

**DOI:** 10.1371/journal.pone.0304132

**Published:** 2024-06-06

**Authors:** Muhammad Azizur Rahman, Tripti Kohli

**Affiliations:** Cardiff School of Technologies, Cardiff Metropolitan University, Llandaff Campus, Cardiff, Wales, United Kingdom; Mattu University, ETHIOPIA

## Abstract

International students’ mental health has become an increasing concern in recent years, as more students leave their country for better education. They experience a wide range of challenges while studying abroad that have an impact on their psychological well-being. These challenges can include language obstacles, cultural differences, homesickness, financial issues and other elements that could severely impact the mental health of international students. Given the limited research on the demographic, cultural, and psychosocial variables that influence international students’ mental health, and the scarcity of studies on the use of machine learning algorithms in this area, this study aimed to analyse data to understand the demographic, cultural factors, and psychosocial factors that impact mental health of international students. Additionally, this paper aimed to build a machine learning-based model for predicting depression among international students in the United Kingdom. This study utilized both primary data gathered through an online survey questionnaire targeted at international students and secondary data was sourced from the ’A Dataset of Students’ Mental Health and Help-Seeking Behaviors in a Multicultural Environment,’ focusing exclusively on international student data within this dataset. We conducted data analysis on the primary data and constructed models using the secondary data for predicting depression among international students. The secondary dataset is divided into training (70%) and testing (30%) sets for analysis, employing four machine learning models: Logistic Regression, Decision Tree, Random Forest, and K Nearest Neighbor. To assess each algorithm’s performance, we considered metrics such as Accuracy, Sensitivity, Specificity, Precision and AU-ROC curve. This study identifies significant demographic variables (e.g., loan status, gender, age, marital status) and psychosocial factors (financial difficulties, academic stress, homesickness, loneliness) contributing to international students’ mental health. Among the machine learning models, the Random Forest model demonstrated the highest accuracy, achieving an 80% accuracy rate in predicting depression.

## Introduction

Mental health includes our emotional, psychological, and social well-being, influencing how we think, feel, and act. It plays a crucial role in our ability to manage stress, build relationships, and make decisions. From childhood to adulthood, mental health remains vital. When individuals face mental health challenges throughout their lives, it can lead to changes in thinking, emotions, and behaviour [1]. Often, society prioritizes physical health and external appearances, sometimes neglecting the significance of mental fitness. Yet, maintaining optimal mental health is important for a seamless journey through life’s various stages, from childhood through adolescence to adulthood. Mental health concerns are non-discriminatory, affecting individuals regardless of age, gender, ethnicity, geographic location, or religious background [2].

The mental well-being of international students is a vital area of research, offering crucial insights into potential mental health concerns and targeted treatment solutions. Trinity College Foundation Studies [3] emphasizes the profound significance of mental health for the educational and personal development of foreign students, underscoring the imperative to analyze and support their mental well-being. The WHO [4] noted that foreign students mental health is a serious issue. According to a WHO [4] assessment more than half of foreign students, approximately 45%, have faced mental health issues like depression and anxiety. Various studies, including Kelo et al. [5] and Huang, R. [6], have found that mental illness is usual in foreign students, and can range from moderate to severe. Foreign students experience more mental health issues for a variety of reasons, including acculturative stress, social isolation, academic pressure, financial challenges, language barriers, and racism [7]. According to studies, overseas students encounter more mental health problems than local students [8]. The Pie News [9] reported the percentage of international students who believe they have "bad mental health" is 36%.

Mental health is critically important for international student’s academic success and to live happily in a new country [10–12]. Multiple research studies have demonstrated that mental health issues like stress, depression, and anxiety are commonly found in international students. The student’s life is miserable because of these mental health issues [13].

However, these studies have focused on mental health problems among international students. There is a scarcity of research on the demographic, cultural, and psychosocial factors that affect mental health of foreign students. Additionally, there is a lack of studies on the accuracy and effectiveness of machine learning based methods for predicting mental illness among international students.

Machine learning algorithms possess the extraordinary ability to analyze vast volumes of data, unveil patterns and predict outcomes. This capability significantly enhances our understanding and prediction of mental health outcomes within this diverse population. Machine learning algorithms enable us to identify individuals at risk of developing mental health issues and intervene before conditions worsen. This remarkable potential for early detection and timely treatment significantly benefits international students.

This study focused on the following objectives: a) Analyze data to understand the demographic, cultural factors, and psychosocial factors that impact mental health of international students; b) Utilize machine learning algorithms to predict depression among international students in the UK.

The motivation behind this study is to gain a deeper understanding and address the unique challenges faced by international students. Common difficulties they encounter, such as adjusting to a new community, language barriers, academic pressures, financial constraints, and social isolation, significantly impact their mental health. By understanding these challenges, new initiatives can be developed to enhance the experiences of international students. The application of machine learning to predict mental illness among international students has the potential to revolutionize the approach to mental health issues within this population. The ability to identify individuals at risk and intervene before conditions worsen through machine learning algorithms could lead to early detection and treatment of mental health problems, ultimately benefiting international students. Therefore, this study on international students’ mental health, utilizing machine learning, has the potential to enhance their well-being through early detection.

## Literature review

The first study on international students’ mental health by Church [14] initiated discussions on their psychological adjustment and unique challenges. Psychological well-being, measured through multiple indicators, significantly impacts resilience. Increasing numbers of students suffering from psychological distress are reflected in university counseling facilities. Mental health issues associated with academic life are frequently reported, with anxiety and depression peaking towards the end of the semester [15]. Research indicates that international students tend to have poorer mental health compared to their peers and their psychological well-being may worsen during university courses [16, 17]. However, some studies lack peer comparison data and fail to explore key mental health factors during university education.

### Depression and anxiety

Moving to a new country for studies can often lead to depression and anxiety among students, with research highlighting depression as the most common clinical symptom among international students seeking university counseling [18]. Sociocultural anxiety has been found to be strongly associated with depression, and various studies have emphasized the relationship between sociocultural anxiety and an increase in depression [13, 19]. Understanding that each student’s experience is unique, researchers have explored the connection between international students and anxiety and depression disorders. Fluency in the English language has been identified as a potential risk factor, with limited English proficiency increasing the likelihood of isolation and exacerbating depression and anxiety [20, 21]. Additionally, a self-identity gap and the ability to express oneself have been identified as crucial factors in the mental well-being of international students. While some Asian students may struggle to differentiate between emotional distress and physical problems, it is important to note that proficiency in the English language varies among individuals [22]. Therefore, not all overseas students may align with the findings of previous studies, necessitating a comprehensive understanding of the diverse experiences of international students.

### Machine learning

We examined several research papers that utilized machine learning algorithms for predicting mental health outcomes. Konda et al. [23] provided evidence for the efficacy of machine learning algorithms in the early-stage prediction of mental health problems. Their study employed various algorithms, including Logistic Regression, KNeighbours classifier, Decision tree classifier, random forests, and stacking algorithms, achieving accuracies exceeding 79%. The use of the Receiver Operating Curve (ROC) analysis further supported the model’s performance, with an area under the curve ranging from 0.8 to 0.9. Although the results indicate the potential of machine learning, it is important to consider the generalizability of these findings and the need for replication in diverse populations.

Shafiee and Mutalib [24] investigated mental health problems among higher education students, highlighting financial problems and the learning environment as significant factors. Their study employed supervised machine learning algorithms, including Random Forest, NB, SVM, KNN, DT, and XGBoost, achieving accuracies ranging from 70% to 96%. While the accuracy rates are promising, the lack of assessment regarding the model’s generalizability and external validity raises concerns. Future research should aim to validate these findings across different student populations and consider potential confounding variables that might affect mental health outcomes.

Hou et al. [25] explored the relationship between reading habits and depressive tendencies among university students using a combination of university library records and mental health questionnaires. By comparing different text categorization algorithms, including kNN, SVM, and naive Bayesian classifier, the study achieved an accuracy of 0.823 using a SVM algorithm. While the findings provide insights into the potential association between reading habits and depressive tendencies, it is crucial to acknowledge the limitations of relying solely on library records and self-report questionnaires, as they may introduce biases and measurement errors.

Spyrou et al. [26] focused on identifying depressive symptoms in elderly participants with cognitive decline, utilizing data mining techniques to analyze electroencephalographic (EEG) data. The study recorded EEG data from participants with geriatric depression and cognitive impairment, employing synchronization analysis through the Orthogonal Discrete Wavelet Transform. Classification using data mining techniques, such as Random Forest, Multilayer Perceptron, and Support Vector Machines (SVM), yielded accuracies ranging from 92.42% to 95.45%.

Ge et al. [27] employed machine learning techniques to predict probable posttraumatic stress disorder (PTSD) in young earthquake survivors. By integrating multiple measures taken two weeks after the earthquake, an XGBoost machine learning algorithm was trained, achieving prediction accuracies ranging from 66% to 85%. While the study presents valuable insights into the prediction of PTSD, the specific sample from a single district and the use of self-report questionnaires and non-standardized measures limit the generalizability and reliability of the findings.

### Summary of machine learning algorithms

In summarizing the studies on student mental health utilizing machine learning algorithms, diverse approaches and outcomes were observed. Konda et al. employed Logistic Regression, KNeighbours classifier, Decision tree classifier, random forests, and stacking algorithms, with the stacking algorithm achieving an impressive 81.75% accuracy in predicting mental health problems. Shafiee and Mutalib explored Random Forest, NB, SVM, KNN, DT, and XGBoost, highlighting the Support Vector Machine (SVM) as the most accurate in predicting mental health concerns. Hou et al. assessed kNN, SVM, and naive Bayesian classifier, finding K-Nearest Neighbors (KNN) with a 76.6% accuracy, SVM with 82%, and Naive Bayes with 64.2%. Spyrou et al. utilized Random Forest, Multilayer Perceptron, and SVM, showcasing a remarkable 95.45% accuracy for Random Forest, slightly lower for MLP at 92.42%, and equally 92.42% for SVM. Ge et al. applied XGBoost, achieving a predictive accuracy of 74.476% for post-traumatic stress disorder, with an AUC of 0.80, indicating its efficacy in distinguishing individuals with probable PTSD. Overall, these studies underscore the diverse applications and notable accuracies of machine learning algorithms in predicting mental health outcomes among students.

## Conclusion

Through the literature review, we discovered several psychosocial factors that are typical among students, such as homesickness, cultural shock, academic stress, and financial difficulties. Additionally, we also uncovered some of the classification machine learning models that are frequently used in research papers, such as Decision Tree, Random Forest, Logistic Regression, K Nearest Neighbor, and SVM. SVM demonstrates good outcomes across various research papers, reaffirming its effectiveness and reliability as a powerful predictive algorithm.

## Methods

### Data source

In our research, we utilized two distinct datasets. The first dataset, gathered through a cross-sectional study conducted in the UK, involved 87 international students who responded to a survey administered via Google Forms (link: https://forms.gle/uatZqx7GwGJrsmXt7) between February and March 2023. We employed a convenience sampling method for data collection. With this dataset, we achieved the first objective of our research, which was to analyze data to understand the demographic, cultural factors, and psychosocial factors that impact mental health of international students. The second dataset, titled ’A Dataset of Students’ Mental Health and Help-Seeking Behaviors in a Multicultural Environment’ [28], was obtained from an online source. The questionnaire used for the survey was designed using elements from 4 standard measurements: Patient Health Questionnaire (PHQ-9), Acculturative Stress Scale for International Students (ASSIS), Social Connectedness Scale (SCS), and General Health Help-Seeking Questionnaire (GHSQ) [28]. This dataset included international student’s mental health information from 201 international students. We specifically used this dataset to apply machine learning algorithms for the purpose of predicting depression among international students.

### Study variables

In this research, objective 1 focused on assessing depression and anxiety as the outcome variables, while objective 2 specifically examined depression, categorized as student has depression (coded as 1) and student does not have depression (coded as 0) across all the models. The features used in this study include demographic, psychosocial and cultural variables for both datasets. In the primary dataset demographic variable include age, gender (Male or Female), relationship status (Yes or No), english proficiency (Beginner, Intermediate, Proficient), education expenses (Loan from bank, Family providing financial support, Self-paying, Other). Psychosocial variables include overwhelmed (Sometimes, Often, Almost always, Never, Rarely), depressed ((Sometimes, Often, Almost always, Never, Rarely), anxiety (Sometimes, Often, Almost always, Never, Rarely). Cultural variable include belonging in community (Somewhat connected but still adjusting, Not very connected still feeling like an outsider, Strongly connected feels like home, Strongly disconnected and homesick), comfortable in new culture(Extremely comfortable I feel like I belong, Not very comfortable still finding my place, Somewhat comfortable, still adjusting to differences, Strongly uncomfortable, feeling like an outsider). In the secondary dataset demographic variable include gender (Male or Female), age, English proficiency (1,2,3,4,5), Intimate (Yes or No). Psychosocial variables include suicidal thoughts (Yes or No), dep (Yes or No), Depression severity (Min, Mild, Moderate, ModSev, Severe), ToSC (total social connectedness measured by SCS), APD (total score of perceived discrimination measured by ASISS questionnaire), AHome (total score of homesickness measured by ASISS questionnaire), APH (total score of percieved hatred measured by ASISS questionnaire), AFear (total score of fear measured by ASISS questionnaire), AGuilt (total score of cultural shock measured by ASISS questionnaire), ToAS (total score of accultrive stress). Cultrual variable include ACS (total score of cultural shock measured by ASISS questionnaire).

### Dependent variable

The dependent variable of the study was the **DEP.**

### Independent variables

Demographic variables, such as age, gender, English proficiency, Suicide and intimacy, were considered. Psychosocial variables including depression severity, ToSC, ToAS, AHome, APD, ACS, Afear, APH, and AGuilt were identified as potential attributes for predicting depression in international students.

### Operationalization of included variables

#### Dep (Depression)

Due to various challenges international students are more susceptible to experiencing depression [28, 29]. The presence of depression among international students is categorized as "yes" if present and "no" if absent.

#### ToSC

The assessment of individual emotional distance or connectedness was conducted using the Social Connectedness Scale, a tool developed by Lee and Robins Lee et al. [30]. The survey, comprising eight statements, required participants to rate each statement on a 6-point Likert scale, ranging from 1 (Strongly Disagree) to 6 (Strongly Agree). The “ToSC” is calculated as the sum of the scores on the 8 statements, where elevated scores correspond to increased levels of social connectedness. (Minh, et al 2019). The potential range for the Social Connectedness score spans from 6 to 48 [28, 30].

#### ToAS

The Acculturation level was assessed using the Acculturative Stress Scale for International Students (ASSIS) [14]. The scale encompasses seven dimensions, namely Perceived Discrimination, Homesickness, Perceived Hatred, Fear, Culture Shock, Guilt, and Miscellaneous. Participants indicated their responses on a 5-point Likert scale, ranging from 1 (strongly disagree) to 5 (strongly agree) (Minh, et al 2019). Eight numerical variables ("APD," "AHome," "APH," "AFear," "ACS," "AGuilt," "AMiscell," "ToAS") were generated based on the ASSIS questions [28].

#### Intimacy

Intimacy referred to the presence or absence of an intimate partner among international students. If a student has an intimate partner, then it’s categorized as "yes", if a student doesn’t have an intimate partner, it is categorized as "no." [28, 29].

#### Suicide

Suicide in this study denotes whether international student experience suicidal thoughts. If a student does experience suicidal thoughts, it is categorized as "yes" otherwise it is categorized as "no." [28].

### Analytic strategy

Python programming language, specifically version 3.9.13, was employed along with various Python packages to conduct data processing and analysis. Our initial steps involved identifying and addressing duplicate and null values in both the primary and secondary datasets. We detected duplicated values within the secondary dataset and subsequently eliminated them. Following this data cleaning process, we proceeded with the analysis of both datasets. We employed data visualization techniques, including word clouds and various bar graphs on the primary dataset. Data encoding was applied to handle categorical features, particularly ordinal ones, where the order needed to be preserved. Label encoding converted each label into an integer value. Additionally, we explored the covariance matrix, a significant matrix in machine learning that provides information on the correlation between features. Feature scaling was performed to normalize the independent features within a specific range, accommodating variations in values, units, and magnitudes during data pre-processing.

We also used four widely used machine learning algorithms- K- nearest neighbors, Random Forest, Logistic Regression and Decision Tree models to predict depression and compared the results of the best algorithm. These four models were selected based on previous literature review. K-Nearest Neighbors (K-NN) is a fundamental machine learning algorithm. The parameter ’K’ represents the number of nearest neighbors and plays a crucial role in the classifier. It involves determining the optimal value for ’K’ so that the ’K’ closest observations are utilized to predict the value of a given observation. For instance, when ’K’ equals 1, the new data object is assigned to the class of its closest neighbor. The proximity of observations is commonly assessed using Euclidean distance [31, 32]. The fundamental principle of K-Nearest Neighbors (KNN) hinges on calculating distances between the tested and trained data samples to identify their nearest neighbors. K-NN is a simple and intuitive classification algorithm. It was chosen to consider the similarity between data points, it makes sense that students with similar demographic, cultural, and psychosocial factors are more likely to have similar mental health outcomes. K-NN was sensitive to local patterns in the data, which was useful for the research. Random Forest is a versatile machine learning method capable of tackling both classification and regression tasks. It operates within the framework of supervised learning, although it is most applied to classification problems. This is because it assembles multiple decision trees into a ‘forest’ and supplies them with random subsets of features from the input dataset, earning it the name random forest [33]. It is robust and often works well even with complex, high-dimensional data. It can capture non-linear relationships and interactions between features, which was essential when studying mental health, as it’s influenced by a different factor. Logistic Regression is a supervised machine learning algorithm. Its role is to forecast a particular outcome variable from an array of independent variables. Logistic regression specializes in predicting the outcomes of specific structured variables, yielding categorical or discrete results. These outcomes may take forms such as 0 or 1, Yes or No and true or false. However, instead of producing exact values like 0 or 1, logistic regression provides probabilistic values that fall within the range of 0 to 1 [23]. Decision tree classifier is the widely used supervised machine learning technique that is used in data mining. A decision tree is a diagram that individuals use to illustrate a statistical likelihood or to determine the sequence of events, actions, or outcomes [24].

### Imbalance data handling

In classification problems, it’s common to encounter datasets where the number of observations for one target class label is significantly lower than those for other class labels. Such datasets are often referred to as "imbalanced class datasets" and are frequently encountered in real-world classification scenarios [34]. As an example, in this study, the target variable is binary: "Yes" (indicating an international student with depression) and "No" (indicating an international student without depression). However, the original dataset exhibited an imbalance, with 49 records representing the ’Yes’ class and 88 records for the ’No’ class. This imbalance can lead to inaccurate and biased predictive outcomes in machine learning.

To address this issue, various techniques, such as oversampling, under sampling, and the synthetic minority over-sampling technique (SMOTE), exist. Oversampling involves replicating minority class data points, while undersampling randomly removes rows from the majority class. In our case, we chose not to explore oversampling and undersampling methods due to their potential drawbacks, such as introducing redundancy or introducing bias and risking the loss of critical information from the majority class.

Instead, we opted for SMOTE, a technique that synthetically generates new instances from existing data. SMOTE focuses on the minority class examples, selecting a random nearest neighbor using the k-nearest neighbor method and creating new synthetic instances in the feature space. In our application, SMOTE proved effective in handling the imbalanced dataset. By leveraging this technique, we generated 39 additional records for the minority class, resulting in a balanced dataset with equal records (88 each) for both ’Yes’ and ’No’ classes. This approach ensures that the classifier assigns equal importance to both classes, mitigating the challenges associated with imbalanced datasets [35]. The overall model performance improved, and accurate results were achieved.

### Model evaluation

In machine learning approaches, the dataset undergoes a random split into two parts: a training dataset, which train the model; a test dataset, used for predicting the response variable and assessing the model’s accuracy against actual outcomes; and a validation dataset which is utilized for estimating parameters incorporated into the training models. In this study, we randomly selected and trained on 70% of the entire dataset subsequently employing 10 fold cross validation. The remaining 30% of the randomly selected data served as our test dataset, where we made predictions to evaluate model performance. We calculated model accuracy metrics, including sensitivity, specificity, positive predictive value, and negative predictive value, to demonstrate the models’ performance in predicting presence or absence of depression among students.

- **True Positive (TP)**: This occurs when the model accurately predicts a positive response outcome.
- **False Positive (FP)**: This happens when the model incorrectly predicts a positive response outcome.
- **True Negative (TN)**: This scenario arises when the model correctly predicts a negative response outcome.
- **False Negative (FN)**: This takes place when the model incorrectly predicts a negative response outcome.
- **Accuracy**: The accuracy of model ranges between 0 to 100 percent accuracy
  **Accuracy = (TP +** **TN)/ (TP + FP + FN+ TN)**
- **Sensitivity**: Sensitivity is a measure of how well the test correctly identifies positive events out of the total number of actual positive events. It quantifies the accuracy of positive predictions among all actual positive cases.
  **Sensitivity = True positive/ (True Positive +** **False Negative)**
- **Specificity**: Specificity represents the proportion of actual negative cases that are correctly predicted as negative. It indicates that there will be another portion of actual negative cases that are incorrectly predicted as positive, termed as false positives.
  **Specificity = True negative/ (True negative +** **False Positive)**
- **Precision**: Precision, also known as positive predictive value, is the ratio of correct positive predictions to the total number of positive predictions made by the classifier [36].
  **Precision = True positive/ (True Positive +** **False Positive)**

To evaluate the model’s capability to differentiate between depressed or not depressed students, we have used the area under the curve (AUC) and receiver operating characteristic (ROC) curve. The ROC curves were employed to compare sensitivity versus specificity across a range of values, providing insights into the model’s ability to predict a binary outcome. The AUC, a summary measure derived from the ROC curve [37], quantifies a classifier’s capacity to distinguish between different classes. As such, a higher AUC corresponds to enhanced model performance in discerning between positive and negative classes [37].

## Results

### Data analysis

According to the data analysis on our primary dataset it was found that financial difficulties, academic stress, homesickness, loneliness, and culture shock were the top factors affecting students’ mental health. It is essential to address these issues to offer international students the necessary assistance they require to maintain their well-being.

In the analysis it was found that the students in the age groups of 21–25 and 26–30 tend to experience higher levels of depression and anxiety. This suggest that these age ranges might be more vulnerable to mental health challenges. Furthermore, delving deeper into the analysis, we uncovered that female students and unmarried students face a higher risk of experiencing depression and anxiety. These findings provide valuable insights into the specific demographic groups that may require targeted support and interventions to promote their mental health and well-being.

Surprisingly, the analysis, failed to uncover any substantial revelations. It became evident that even students who possessed a high level of proficiency in English were still grappling with mental health challenges. This led us to a compelling conclusion that English language skills should not be seen as a barrier to addressing and supporting students’ mental well-being. It suggests that factors beyond language fluency play a more crucial role in tackling mental health issues among students. These findings encourage us to adopt a holistic approach that encompasses various aspects of student well-being beyond language proficiency alone.

Furthermore, the data analysis on the primary dataset revealed that students who have taken loans for their studies are more likely to experience depression and anxiety.

Regarding cultural factors, we discovered that students that are adjusting to a new culture, encounter less depression and anxiety. This finding was encouraging, as it suggests that institutions can take measures to facilitate the cultural adjustment process for international students.

### Predicting depression

In Table 1 below, we present the results of four machine learning models: K Nearest Neighbour (K-NN), Random Forest (RF), Logistic Regression (LR), and Decision Tree (DT). The accuracy of depression prediction was generally modest for all models, ranging from 50% to 65% on the test dataset. Notably, the Random Forest model achieved the highest overall accuracy. Although the Random Forest model exhibited low sensitivity, indicating less precision in identifying students without depression, it demonstrated high specificity, excelling at identifying students with depression. Specifically, the Random Forest model correctly identified 97% of students without depression (33 out of 34) and 23% of students with depression (6 out of 26). This suggests that the RF model showed relative strength in predicting both absence and presence of depression in international students. Conversely, Logistic Regression, K-NN, and Decision Tree models exhibited lower overall accuracy (58%, 57%, and 50% respectively), along with reduced sensitivity, specificity, and positive and negative predictive values.

**Table 1:** Confusion matrix.

Confusion Matrix		K-NN		RF		LR		DT	
		Predicted		Predicted		Predicted		Predicted	
		No Dep	Dep	No Dep	Dep	No Dep	Dep	No Dep	Dep
**Observed**	**No Dep**	**34**	0	33	1	34	0	28	6
	**Dep**	**26**	0	20	6	25	1	24	2
**Accuracy**			57%		65%		58%		50%
**Sensitivity**			56%		62%		57%		53%
**Specificity**			0%		86%		100%		25%
**Positive predicted value**			100%		97%		100%		82%
**Negative predictive value**			0		23%		3.8%		7.7%
**AUC**			70%		84%		76%		49%

**Fig 1:**
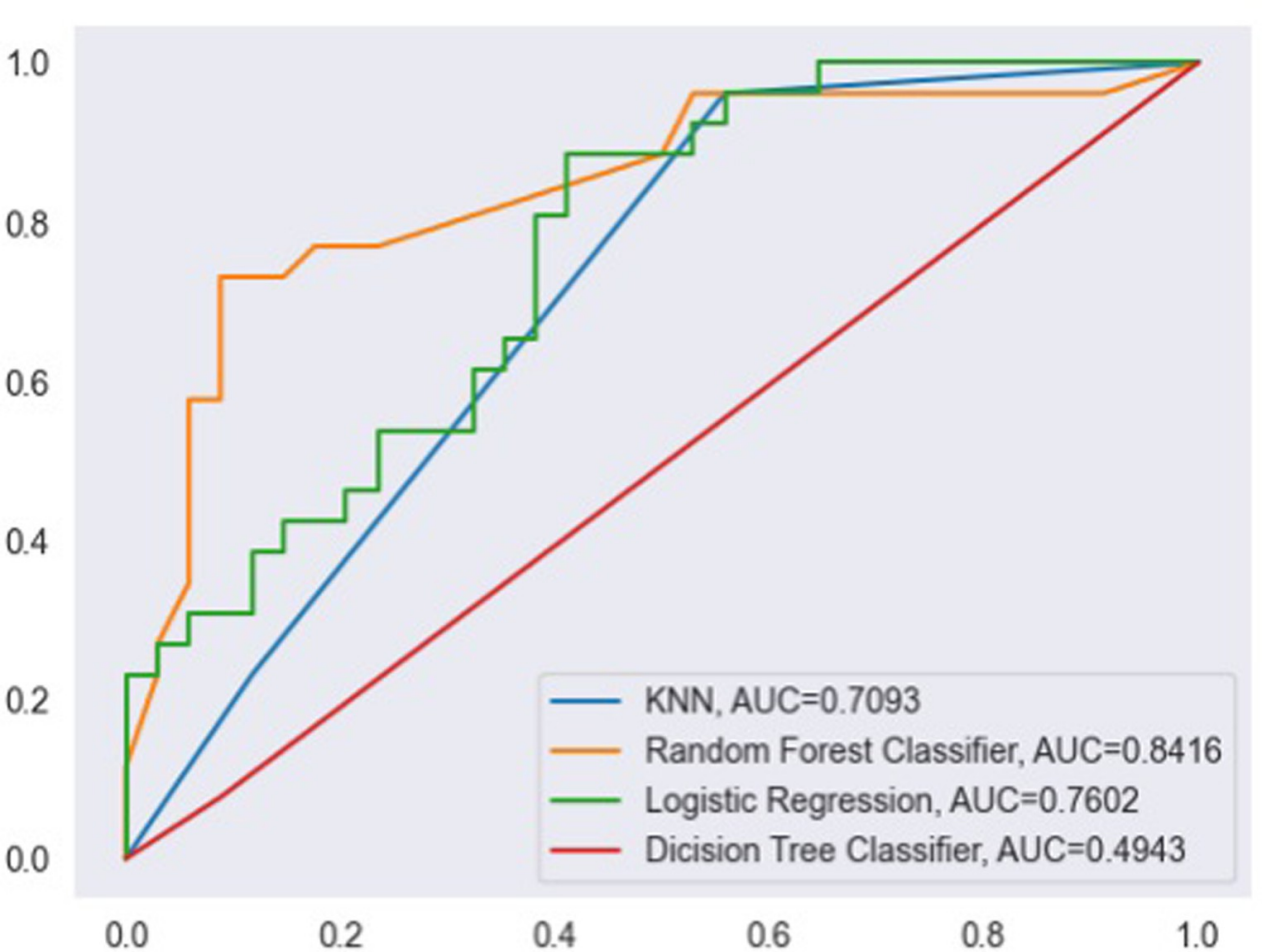
ROC curves for four models.

Below Fig 1 displays a visualization of the receiver operating characteristics (ROC) curve. Among the four machine learning models utilized in this research, the ROC curve of the Random Forest model exhibits the highest AUC value, signifying its superior performance in classifying presence and absence of depression in international students when compared to the other models.

### Important attribute to predict the depression in the international students

**Fig 2:**
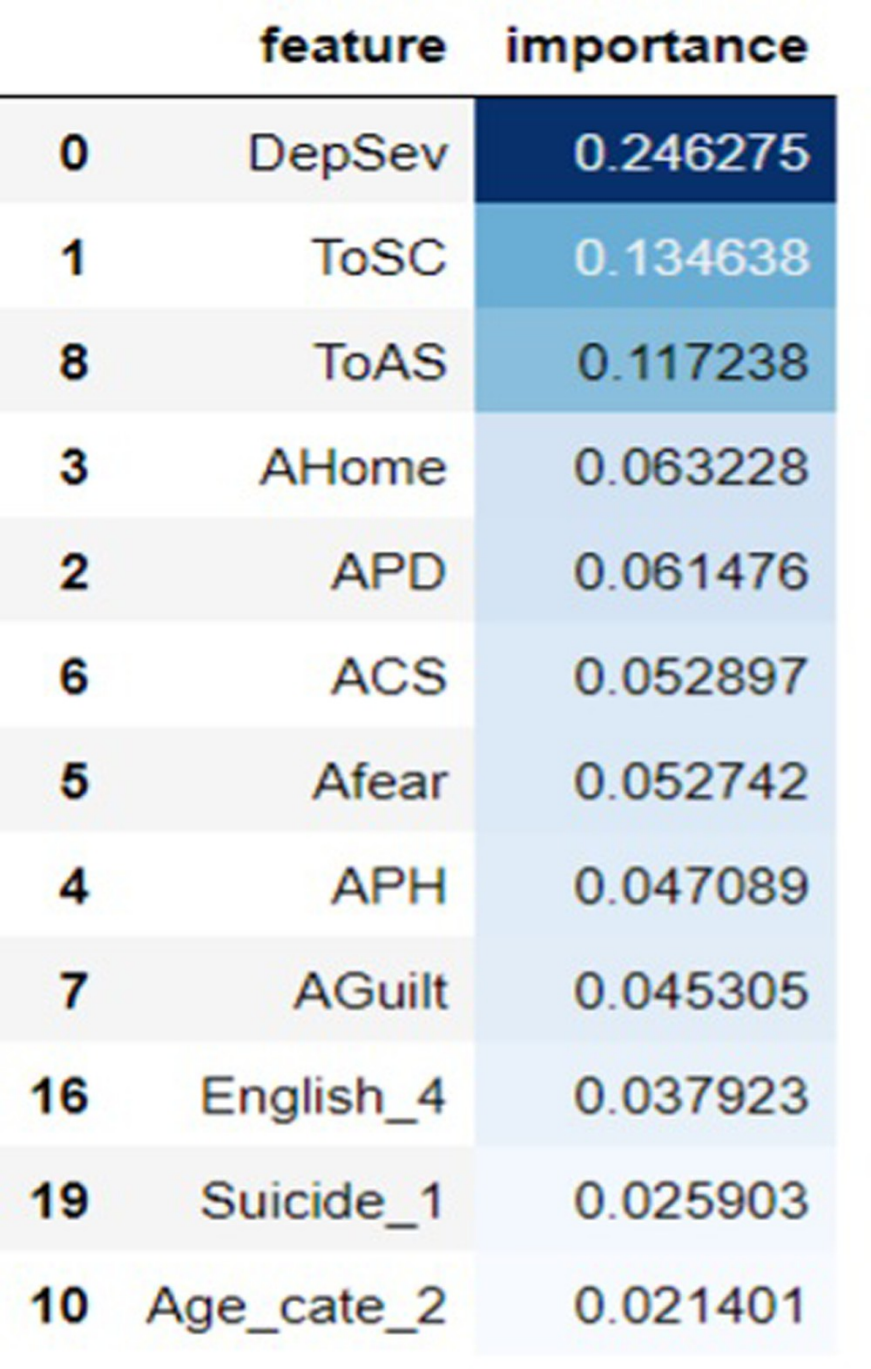
Important feature.

Key attribute of depression in international students were assessed using gini coefficients within a 10-fold cross-validation framework. To identify significant predictors, we considered the top performing model (random forest). As a result, the leading factors contributing depression among international students were found to be ToSC (Social Connectedness), ToAS (Accultrive Stress), AHome (Homesickness), APD (Perceived discrimination), ACS (Cultural Shock), AFear (Fear), APH (Perceived Hatred), AGuilt (Guilt). Further essential attributes associated with depression in international students are detailed in Fig 2.

## Discussion

In addressing the first objective of this study, we conducted a comprehensive analysis of demographic, cultural, and psychosocial factors influencing the mental health of international students in the UK. The primary dataset, obtained through a cross-sectional survey, highlighted various significant determinants. Financial difficulties, academic stress, homesickness, loneliness, and culture shock emerged as prominent factors impacting students’ mental health.

The demographic analysis revealed noteworthy patterns with certain age groups and gender categories exhibiting higher vulnerability to depression and anxiety. Students in the age groups of 21–25 and 26–30 displayed higher levels of mental health challenges. Additionally, female and unmarried students were identified as groups at an elevated risk. This finding is also supported by few studies that student in their 20’s, female and unmarried students are most prone to have mental health challenges [10, 28, 29]. The present data analysis indicated that English proficiency appeared to have a relatively lower importance in predicting depression and anxiety. However, it’s worth noting that other research [21], has emphasized the crucial role of English proficiency in mental health.

Moreover, the revelation that students relying on loans for their studies are more likely to experience mental health issues emphasizes the intricate relationship between financial stress and mental well-being. This finding was supported by several studies [7, 10, 21, 38]. Cultural factors also played a role with students in the process of adjusting to a new culture reporting lower levels of depression and anxiety. This conclusion was substantiated by analogous findings in other research [29, 39, 40]. This finding suggests that facilitating cultural adaptation processes can contribute positively to students’ mental health.

In this study, the second objective was to predict depression among international students using machine learning algorithms. We implemented four machine learning algorithms such as K-Nearest Neighbors, Random Forest, Logistic Regression, and Decision Tree models. The model performance of each of the four supervised machine learning algorithm was compared by their classification accuracy and AUC score values. According to the analysis report, the sensitivity and AUC value of the random forest model was 65% and 84% with 10-fold cross-validation, respectively. Hence, the random forest algorithm model was the most accurate model to predict depression in international student.

The Receiver Operating Characteristic (ROC) curve further illustrated the Random Forest model’s robustness, displaying the highest Area Under the Curve (AUC) value compared to other models. This emphasizes its effectiveness in classifying the presence and absence of depression. We also identified key attributes contributing to depression among international students, including social connectedness, acculturative stress, homesickness, perceived discrimination, cultural shock, fear, perceived hatred, and guilt. Notably, the significant impact of several of these factors on depression is discussed in existing literature [8, 12–14]. This is also consistent with the finding conducted on mental health of employees in technical and non-technical companies [2].

In conclusion, our study not only unveiled critical factors influencing the mental health of international students but also demonstrated the potential of machine learning models, particularly the Random Forest algorithm, in predicting depression. These findings contribute valuable insights for institutions and policymakers aiming to enhance the mental well-being of international students.

### Implications

The implications of this study are significant as it highlights the need to prioritize mental health assistance for international students. Institutions can use the result of this research to develop appropriate interventions to support students who have a greater risk of developing mental health problems. The research can also help to shape policies aimed at providing adequate resources and support for international students.

## Conclusion

According to the findings of this study, demographic, cultural, and psychosocial elements have an important impact on the international student’s mental health. The most significant factors affecting mental health were discovered to be financial difficulties, academic stress, homesickness, loneliness, and culture shock. This study also found that students aged 21–30, female students, and unmarried students experience more depression and anxiety. It was also found that English proficiency is not a significant factor in mental health issues. This study has also developed a machine learning model that can be used to predict depression among international students. Four models on the secondary dataset were implimented, and they are Logistic Regression, Decision Tree, Random Forest, and K Nearest Neighbor, compared all the models based on accuracy, classification report, ROC and AUC score, and found that the Random Forest outperformed the other models.

## Recommendation

Based on the findings, this study recommends that institutions providing education to international students should prioritize addressing financial difficulties, academic stress, homesickness, loneliness, and culture shock. Additional support systems and resources should be made available to students who are more likely to suffer from mental health problems, such as students between the ages of 21–30, female students, and unmarried students.

### Limitations & future work

This study is subject to a limitation due to the relatively small amount of data available for analysis. Although both primary and secondary data sources were utilized both have a smaller number of records. As a result, caution should be exercised when interpreting the findings of this research.

Another limitation is that we had limited time to collect and analyze data. We were not able to gather a lot of information and study the factors affecting the mental health of international students in depth. This limitation might have prevented us from exploring important factors or understanding all the nuances that could affect our findings.

To overcome these limitations, future research should use a larger dataset with more participants to get more reliable and representative results. By including a wider range of people, future studies can better understand how mental health problems are influenced by different factors like demographics, cultural, and psychosocial factors among international students. Also, allocating enough time for data collection and analysis will allow us to thoroughly study the topic and consider other variables that might affect mental health outcomes.

## Supporting information

S1 Dataset Primary dataset. (CSV)

S2 Dataset Secondary dataset. (CSV)
